# Getting Better or Worse? General Health Status of 9^th^ Grade Students in Orhangazi, Bursa, Turkey

**DOI:** 10.1100/tsw.2008.50

**Published:** 2008-03-25

**Authors:** Zuleyha Alper, Hakan Ozdemir, Yesim Uncu, Alis Ozcakir, Ganime Sadikoglu, Nuran Bayram

**Affiliations:** ^1^ Faculty of Medicine, Department of Family Practice, University of Uludag, 16059 Bursa, Turkey; ^2^ Faculty of Economics and Administration, Department of Econometrics, University of Uludag, 16059 Bursa, Turkey

**Keywords:** students, general characteristics, eating attitudes, physical activity, smoking, depression

## Abstract

Adolescence is a transition phase from childhood to adulthood. In this period, rapid changes and development in their physical, biological, psychological, and social lives take place. While adolescents have to acquire many qualifications, they are faced with many problems, especially those that risk their health. In Turkey, one of the most important issues contributing to risky behaviors is the 1^st^ Phase Nationwide High School Exam. Students must pass this phase in order to be in good high schools and to then pass the 2^nd^ Phase University Exam. Most of their time is spent studying in school or in private teaching institutions, and less time is spent with their families or participating in social activities. In order to examine the effects on 9^th^ grade students after the 1^st^ Phase exams, we conducted this study with 1192 students in Bursa, Orhangazi. Data to evaluate students by socioeconomic status, body mass index (BMI), dietary, smoking, and physical activity behaviors and psychological status were collected via classroom questionnaires. We aimed to determine and evaluate the general characteristics and physical examination findings, to some extent, in a nationally representative sample of 9^th^ grade students a year following the Nationwide High School Exam.

## INTRODUCTION

The psychological and social health levels of children and young people have a direct impact on society's health level and productivity. Improvement in health care can be obtained by ensuring that individuals practice behaviors that leverage their well-being and reduce the risk of diseases. Healthy behaviors are those that people believe are beneficial and practice to improve their health status and prevent the onset of diseases. Adolescence is the period when rapid changes and developments take place in physical, biological, psychological, and social status, and is recognized as a transition phase from childhood to adulthood. During adolescence, young people acquire many qualifications; on the other hand, they also face many problems and behave in ways that risk their health the most. The period from 15 to 17 years of age is the time of gaining independence and choosing a lifestyle. According to the health improvement matrix, positive healthy behaviors include not smoking, healthy nutrition, physical activities, sexual consciousness, avoiding accidents, not having a drug or similar substance dependence, oral hygiene, self-development, productivity, handling stress, regular health examinations, and behaving in favor of hygiene improvement[1,2,3,4,5]. According to population statistics in 2000, there were 14.1 million adolescents between the ages of 10 and 19 out of the total population of 67.5 million in Turkey[6].

Adolescents experience the dilemma of gaining independence from their families, but needing the support and care of their families as well. Adolescents experience loneliness and a lack of confidence and power while they are getting away from their families. These emotions may further lead to feelings of desperation and psychological breakdown, and even to behaviors that jeopardize their health. The risky behaviors include unhealthy weight management measures, interpersonal communication problems within the family, failure in school, examination stress, suicide attempts, and decline in oral hygiene (i.e., teeth brushing). This is also a period of new experiences[4,7,8]. Adolescents tend to try alcohol, smoking, and drugs. Approximately 62.8% of males and 24.3% of females, and 43.6% of the total population older than 15, smoke in Turkey. One out of five children between 13 and 15 also smoke. Every day, 80,000–100,000 new children start smoking all over the world, and 50% of people who started smoking during adolescence kept their habits for 15–20 years[9,10,11,12].

Well-balanced nutrition is of the utmost importance for adolescents who are in fast-growing and developing ages. Dairy products and yoghurt, meat or legumes, and fresh fruits and vegetables should be consumed every day. Fresh fruit and vegetable consumption is low, particularly among males. The most frequent nutritional misbehaviors include eating fast food and skipping meals. Breakfast is generally the most skipped meal. Eating disorders, voluntary vomiting, and diuretic and laxative abuse are prevalent among female adolescents[13].

We can see a great deal of difference in health problems among students. Health problems among school children show diversity between different countries. A young person in a phase of personality formation is prone to many changes; hence, it is wise to consider carefully all the kinds of changes that take place in our country, and worldwide in general, in order to evaluate the condition of school children properly[4,14]. Turkey is a country with significant differences among regions and socioeconomic status. This situation is an important factor that shapes health problems specific to students and schools[14].

There has been a relative improvement in welfare in Turkey. However, aspirations have also risen, and the differences between social groups in settlements from countryside to urban areas became manifest. Unemployment and fractured families led the young population to leave their education and work as cheap labor. Due to shifts and extended working hours, parents led irregular lifestyles, which ultimately brought cheap and unhealthy food and cheap and unhealthy TV programs into their children's lives. Extended TV watching time damaged the quality and frequency of relationships between family members. Reduced family relationships lead to early sexual experiences, early and unhealthy diets, and unnecessary esthetic concerns[7].

Turkey has another important issue that plays a significant role in elementary and high school students' lives: the 1$^{st}$ Phase Nationwide High School Exams and the 2$^{nd}$ Phase University Exams. These exams are supposed to select successful students; however, only a very limited number of young people (10% of students who take university exams) are lucky enough to pass and enroll. The aforementioned exams have shaped the education system in Turkey and anything unrelated to these exams in the system became valueless. High school exams in the 1$^{st}$ phase isolated 8$^{th}$ grade students from daily life and became an important issue for them. On the other side, 1$^{st}$ phase exams cause other problems: students spend their time in school, private teaching institutions, and private lessons; they focus on exams; they spend less time with their families; and they quit well-balanced diets and sports activities. These children are defined by our Minister of Education as "a generation of eating toast and having test"[15].

This study was done to evaluate the possible changes in lifestyle, psychological status, and daily habits in 9$^{th}$ grade students in the Orhangazi district of Bursa — which has an economy that depends on agriculture and industry — who had just experienced the burden of 1$^{st}$ phase exams.

## MATERIALS AND METHODS

This study included 9$^{th}$ grade students in five high schools during the 2005–2006 educational year in Orhangazi, Bursa. Permission from the “Regional Directorate of Education” in Orhangazi was obtained to screen the students in the schools. The schools were informed accordingly. Data collection was done in cooperation with the last-year students of the University of Uludag, School of Medicine, who were in their internship period in the Family Medicine Department. Medical students were trained about the surveys and the physical examinations before initiating the study. Surveys consisting of four parts were given to the students according to the schedule given to the schools. The first part of the survey covered the following information: students’ age, gender, educational status and occupation of the parents of the student, monthly income and the social security coverage of the parents, number of brothers and sisters, social competence of the student according to the teachers, and the weighted success rate of the student in the first semester. The second part asked about the following information: dietary habits of the student, physical activities, and smoking habits. The Garner and Garfinkel Eating Attitude Test (EAT-40) and the Kovacs Children’s Depression Inventory (CDI) were implemented[16,17]. The study was concluded with weight and height measurements and eye, nose, throat, skin, and hair examinations. However, if the student suggested any medical problem, he/she was invited to our outpatient clinics, detailed physical examinations were conducted, and necessary treatments were planned or advised for further diagnosis and treatment. Examinations were done under the supervision of lecturers from the Family Medicine Department. Appropriate treatment was suggested for students with detected pathologies. In case of second-line diagnostic or treatment need, students were advised to have a further medical consultation with a written report.

### Scales

The EAT-40 was originally used for the purpose of determining the eating behaviors and approach of anorexia nervosa patients. This test is also used as a screening test for a normal individual’s potential problems in eating behaviors. Developed by Garner and Garfinkel, the reliability and validity analysis process was done by Savaşır and Erol in Turkey[18]. The test is useful for those persons already clinically defined as “patients” and to determine those “normal” persons who may have the potential for such disorders.

The Kovacs CDI was used to assess depressive symptoms. It is a well-validated and widely used instrument that was developed to measure symptoms of depression within the community. The scale is valid for use in student populations. Reliability and validity analyses were done in Turkey by Öy[19]. Positive scores higher than 19 should be considered indications of depression.

Standard BMI criteria developed for Turkish children were used to assess BMI of the study group[20]. Obesity was defined as a BMI (kg/m$^2$) greater than or equal to the 95$^{th}$ percentile for age and gender; overweight as a BMI greater than or equal to the 85$^{th}$ percentile, but less than the 95$^{th}$ percentile; normal as a BMI between the 15$^{th}$ and 85$^{th}$ percentiles; and the rest defined as having low BMI. Because the 95$^{th}$ percentile BMI in the reference growth charts was >30.0 in girls of 15 years of age and in boys of 16 years of age, a BMI of 30 or higher was considered obese in accordance with standard adult criteria. Weight and height measurements of the study group were taken by researchers, but student’s parents’ weight and height measurements were obtained by students’ declarations and those BMI were calculated by researchers according to standard adult criteria.

Vision examinations were performed using the Snellen scale, and color blindness was examined using the Ishihara scale. Students who could not see at least two of the letters in the bottom line were accepted as having vision problems. Two indicators of sedentary behavior were obtained by the questions “on school days how many hours you usually spend by watching TV” and “on school days how many hours you usually spend with PC.”

The data from the surveys and examination reports were entered into the computer, and the relationships between the classified variables were analyzed by using the Statistical Package for Social

Sciences (SPSS, Chicago, IL, version 11.0). Descriptive statistics were used to assess general characteristics of the study group. Differences in proportions for categorical variables were tested by Pearson Chi-square and Cramer's V. Statistical significance was defined as $p < 0.05$.

## RESULTS

All 9th grade students in five high schools in Orhangazi, Bursa, were included in the study (N = 1192). Of the students, 652 were male (54.7%) and 540 were female (45.3%). Of that total, 886 (74.3%) of the students could be reached, although the visits were in line with the prescheduled timetable shared with the schools. The students who could not be reached were those who were absent on the physical examination day. In the end, the study group consisted of 474 males (53.5%) and 412 females (46.5%). The students were between the ages of 14 and 20 (mean age 15.24 ± 0.53).

According to the results of the present study, 6.0% of the mothers and 0.8% of fathers were illiterate, approximately 25.3% of the study group had no social security coverage, and we found that 45.8% had low-income degrees according to data from the Turkish Statistical Institute[6]. The sociodemographic characteristics of the study group are summarized in Table 1.

The ratio of the students who had social adaptation problems in the classroom, according to their teachers, was 4.7%. There was no statistical significance according to gender ($p > 0.05$), but students with high depression scores ($p < 0.05$) or living with an elderly grandparent at home ($p < 0.05$) had many more adaptation problems within classrooms. The first semester success rates demonstrate that female students were significantly more successful than male students ($p < 0.001$), and students who had excellent semester rates had no adaptation problems within classrooms ($p < 0.001$). Comparison of variables according to gender is shown in Table 2.

Table 3 summarizes the evaluation of the students according to their BMI: 57.8% of the students were normal, 32.0% were underweight, 7.8% were overweight, and 2.4% were obese. We found that 22.7% of students of the low BMI group and 1.5% students of the obese groups had mothers who had an educational degree of primary school.

The BMI of the parents show that 30.4% of the mothers were overweight and 19.6% were obese; 35.3% of fathers were overweight and 13.7% were obese. Although students of overweight and obese mothers were much more likely to be obese and overweight, this relationship is not statistically significant ($p > 0.05$).

Approximately 15.5% of the students had an eating disorder according to the EAT-40 (Table 4). This ratio was 19.7% in all females and 11.8% in all males ($p < 0.001$). We found a statistically significant relationship between eating disorders and gender, BMI, and semester rates. Female students who had high BMIs ($p < 0.05$) and low semester rates ($p < 0.05$) were much more likely to have high EAT-40 scores ($p < 0.005$). Also, students who had low physical activity hours (1–2 h/week) ($p < 0.005$) and high depression scores on the Kovacs CDI ($p < 0.005$) showed high scores in EAT-40.

In the present study, in addition to 2 h of compulsory physical activity (PA) lessons, we found that 36.2% of the study group had additional PA hours: 25.7% of this group spent 3–5 h, 7.3% spent 6–10 h, and 1.9% spent more than 10 h with PA, especially on sports teams at their schools. Boys were more active than girls ($p < 0.001$) and were more likely to choose football and basketball; while girls were more likely to choose volleyball, gymnastics, and athletic games.

Approximately 83.4% of students knew how to use a PC to some extent, 36.7% of the students had PCs of their own, and the ratios of students and hours spent with the PC on school days were as follows: 48.3%, 1–2 h; 15.6%, 3–5 h; and 10.9%, more than 5 h (Table 2). When the other indicator of sedentary behavior, i.e., hours spent watching TV on school days, was evaluated, we found that 52.9% of students spent 0–2 h, 36.3% spent 3–4 h, 7.7% spent 5–7 h, and 3.1% spent more than 7 h watching TV. According to our results, boys spent more time on PCs ($p < 0.001$) and girls spent more time watching TV ($p < 0.005$). Students who spent more time on PCs substantially belonged to the group who had only compulsory PA lessons ($p < 0.005$).

**TABLE 1**
**Sociodemographic Characteristics of the Study Group**

| | | n | %* |
|---|---|---|---|
| Gender | Male | 474 | 53.5 |
| | Female | 412 | 46.5 |
| Mother's educational status | Illiterate | 53 | 6.0 |
| | Primary school | 596 | 67.7 |
| | High school | 213 | 24.2 |
| | University | 19 | 2.2 |
| Father's educational status | Illiterate | 7 | 0.8 |
| | Primary school | 403 | 46.1 |
| | High school | 378 | 43.2 |
| | University | 87 | 9.9 |
| Mother's employment status | Still working | 125 | 14.1 |
| | No working | 720 | 81.4 |
| | Retired | 39 | 4.4 |
| Father's employment status | Still working | 678 | 78.7 |
| | No working | 61 | 7.1 |
| | Retired | 122 | 14.2 |
| Mothly Income | 0–625$ | 358 | 45.8 |
| | 625–1250$ | 344 | 44.0 |
| | >1250$ | 79 | 10.1 |
| Social Security Coverage | Social Security Ins. | 382 | 44.4 |
| | Self-Employed Fund | 144 | 16.7 |
| | Retirement Fund | 116 | 13.5 |
| | Not any | 218 | 25.3 |
| Adaptation in school | Good | 674 | 95.3 |
| | Bad | 33 | 4.7 |
| School grade | Poor | 65 | 10.4 |
| | Fair | 229 | 36.8 |
| | Good | 240 | 38.5 |
| | Excellent | 89 | 14.3 |
| PC at home | Yes | 325 | 36.7 |
| | No | 561 | 63.3 |

* Column percentages.

When the smoking habits of the study group were evaluated as never smoked, tried but quit smoking, and smoked, the results were as follows, respectively: 87.2, 11.2, and 1.6%. Boys were more likely to smoke than girls ($p < 0.001$). Approximately 58.2% of students declared that at least one of their parents smoked, but we found no statistical significance between parental and students' smoking (Table 5).

According to the Kovacs CDI results, we found that 9.5% of the study group had depressive symptoms (Table 6). Girls ($p < 0.001$), students who had adaptation problems within classrooms ($p < 0.05$), those who had junk food habits ($p < 0.05$), and those who had high scores on the EAT-40 ($p < 0.001$) were more likely to have high scores for depression.

The physical examinations revealed no pathology in 66.1% of the students who were included in the study and the examination results were summarized in Table 7.

**TABLE 2**
**Comparison of Variables According to Gender**

| Variables | Male n | Male %† | Female n | Female %† | |
|---|---|---|---|---|---|
| Adaptation in school | | | | | |
| Yes | 326 | 46.1 | 348 | 49.2 | 1.068* |
| No | 19 | 2.7 | 14 | 2.0 | 0.196** |
| | | | | | 0.039*** |
| School grade | | | | | |
| Poor | 21 | 3.4 | 44 | 7.1 | 34.590* |
| Fair | 119 | 19.1 | 110 | 17.7 | **0.000**** |
| Good | 112 | 18.0 | 128 | 20.5 | 0.236*** |
| Excellent | 16 | 2.6 | 73 | 11.7 | |
| BMI | | | | | |
| Low BMI | 104 | 11.7 | 180 | 20.3 | 52.046* |
| Normal | 306 | 34.5 | 206 | 23.3 | **0.000**** |
| Overweight | 50 | 5.6 | 19 | 2.1 | 0.242*** |
| Obese | 14 | 1.6 | 7 | 0.8 | |
| Daily meals | | | | | |
| 1 | 4 | 0.5 | 3 | 0.3 | 38.902* |
| 2 | 67 | 7.7 | 130 | 15.0 | **0.000**** |
| 3 | 350 | 40.3 | 243 | 28.0 | 0.212*** |
| 4 | 36 | 4.1 | 24 | 2.8 | |
| 5 | 4 | 0.5 | 2 | 0.2 | |
| ≥6 | 3 | 0.3 | 2 | 0.2 | |
| Junk meal | | | | | |
| Yes | 344 | 39.3 | 343 | 39.2 | 13.024* |
| No | 122 | 13.9 | 66 | 7.5 | **0.000**** |
| | | | | | 0.122*** |
| TV watching hours | | | | | |
| 0–2 h/day | 253 | 28.5 | 216 | 24.4 | 16.181* |
| 3–4 h/day | 157 | 17.7 | 165 | 18.6 | **0.003**** |
| 5–7 h/day | 50 | 5.6 | 17 | 1.9 | 0.135*** |
| >7 h/day | 14 | 1.6 | 14 | 1.6 | |
| Junk food while TV watching | | | | | |
| Yes | 290 | 34.3 | 302 | 35.7 | 16.480* |
| No | 163 | 19.3 | 91 | 10.8 | **0.003**** |
| | | | | | 0.140*** |
| PC use | | | | | |
| Yes | 414 | 46.7 | 325 | 36.7 | 11.395* |
| No | 60 | 6.8 | 87 | 9.8 | **0.001**** |
| | | | | | 0.113*** |
| PC playing hours | | | | | |
| 0 h/day | 81 | 9.1 | 142 | 16.0 | 37.346* |
| 1–2 h/day | 245 | 27.7 | 183 | 20.7 | **0.000**** |
| 3–5 h/day | 87 | 9.8 | 51 | 5.8 | 0.205*** |
| >5 h/day | 61 | 6.9 | 36 | 4.1 | |
| Physical activity‡ | | | | | |
| Yes | 218 | 25.3 | 94 | 10.9 | 54.664* |
| No | 240 | 27.9 | 309 | 35.9 | **0.000**** |
| | | | | | 0.252*** |
| Physical activity hours | | | | | |
| 0 h/day | 1 | 0.1 | 2 | 0.2 | 64.716* |
| 1–2 h/day | 258 | 29.1 | 315 | 35.6 | **0.000**** |
| 3–5 h/day | 143 | 16.1 | 85 | 9.6 | 0.270*** |
| 6–10 h/day | 59 | 6.7 | 6 | 0.7 | |
| >11 h/day | 13 | 1.5 | 4 | 0.5 | |

**TABLE 2 (continued)**

| Variables | Male | | Female | | |
|---|---|---|---|---|---|
| | **n** | **%†** | **n** | **%†** | |
| Smoking history | | | | | |
| Nonsmoker | 375 | 42.5 | 394 | 44.7 | 49.833* |
| Exsmoker | 82 | 9.3 | 17 | 1.9 | **0.000*** |
| Regular smoker | 13 | 1.5 | 1 | 0.1 | 0.238*** |
| Kovacs CDI | | | | | |
| Negative | 446 | 50.3 | 356 | 40.2 | 15.169* |
| Positive | 28 | 3.2 | 56 | 6.3 | **0.000*** |
| | | | | | 0.131*** |
| EAT-40 | | | | | |
| Negative | 418 | 47.2 | 330 | 37.3 | 10.483* |
| Positive | 56 | 6.3 | 81 | 9.2 | **0.001*** |
| | | | | | 0.109*** |

† Total percentage.
‡ PA with the exception of school PA hours.
* Pearson chi-square.
** p value (Bold numbers are found to be statistically significant).
*** Cramer's V.

## DISCUSSION

Orhangazi is a town in Bursa on the way to İstanbul, with an economy that depends on agriculture and industry, which attracts residents from rural areas as well. The population of the city is 68,902; 39% of the population works in the textile and metal industry. The working hours in these industries are generally shifts. According to 2005 income distribution rates, 6.1% of the Turkish population has high incomes and 44.4% has low income rates[6]. In our study, 10.1% of students belonged to the high income group. This might be related to the extensive employment opportunities in the Orhangazi district.

Adolescents compose approximately one-third of our population and are of great importance in terms of Turkey's future; they deserve care. School children are the age group least likely to be referred to a health care professional[21]. Not smoking, healthy diets, and regular physical activities are among the prioritized healthy behavioral targets[7]; therefore, all around the world, many studies have been conducted to understand the usual problems that adolescents face.

A study conducted in Bursa that evaluated students aged 6–17 years found that 6.2% of students were overweight and 2.1% were obese[22]. Two other studies from Turkey reported that obesity prevalence was 12.8 and 9.1%, respectively[21,23]. In the U.S., obesity prevalence was found to be 13.7% in girls and 11.7% in boys[24]. In this study, we found 7.8% of children were overweight and 2.4% were obese. The results were consistent with the previous study by Aydin et al., but lower than the other studies[21,22,23].

There were several known factors that effected obesity, such as educational status of parents (especially mothers), monthly income rates, students' degree of physical activity, and hours spent watching TV. There is an adverse correlation between mothers' educational status and obesity. A child's risk of obesity increases with the low educational degree of the mother[25]. In the present study, we found that low educational degree of the mother effected both low BMI and obesity. In our study, we found a higher obesity prevalence in boys (1.6%) and a low BMI prevalence in girls (20.3%), and both of these childrens' mothers were especially from the primary school degree group.

**TABLE 3**
**Distribution of Study Group According to BMI Measurements**

| **Variables** | **Low BMI** | | **Normal** | | **Overweight** | | **Obese** | | |
|---|---|---|---|---|---|---|---|---|---|
| | **n** | **%†** | **n** | **%†** | **n** | **%†** | **n** | **%†** | |
| Age | | | | | | | | | |
| 14 | 7 | 0.8 | 35 | 4.0 | 1 | 0.1 | 1 | 0.1 | 20.872* |
| 15 | 173 | 19.5 | 337 | 38.0 | 55 | 6.2 | 12 | 1.4 | $\mathbf{0.002}^{**}$ |
| ≥16 | 104 | 11.7 | 140 | 15.8 | 13 | 1.5 | 8 | 0.9 | $0.109^{***}$ |
| Gender | | | | | | | | | |
| Male | 104 | 11.7 | 306 | 34.5 | 50 | 5.6 | 14 | 1.6 | 52.046* |
| Female | 180 | 20.3 | 206 | 23.3 | 19 | 2.1 | 7 | 0.8 | $\mathbf{0.000}^{**}$ |
| | | | | | | | | | $0.242^{***}$ |
| Mother's education | | | | | | | | | |
| Illiterate | 16 | 1.8 | 32 | 3.6 | 5 | 0.6 | 0 | 0.0 | 7.031* |
| Primary school | 200 | 22.7 | 339 | 38.5 | 44 | 5.0 | 13 | 1.5 | $\mathbf{0.050}^{**}$ |
| High school | 60 | 6.8 | 130 | 14.8 | 16 | 1.8 | 7 | 0.8 | $0.052^{***}$ |
| University | 6 | 0.7 | 9 | 1.0 | 3 | 0.3 | 1 | 0.1 | |
| Mother's employment status | | | | | | | | | |
| Still working | 48 | 5.4 | 66 | 7.5 | 9 | 1.0 | 2 | 0.2 | 8.535* |
| Housewife | 215 | 24.3 | 430 | 48.6 | 58 | 6.6 | 17 | 1.9 | $0.201^{**}$ |
| Retired | 19 | 2.1 | 16 | 1.8 | 2 | 0.2 | 2 | 0.2 | $0.071^{***}$ |
| Mothly income | | | | | | | | | |
| 0–625$ | 98 | 12.5 | 230 | 29.4 | 22 | 2.8 | 8 | 1.0 | 13.650* |
| 625–1250$ | 119 | 15.2 | 188 | 24.1 | 31 | 4.0 | 6 | 0.8 | $\mathbf{0.034}^{**}$ |
| >1250$ | 23 | 2.9 | 44 | 5.6 | 7 | 0.9 | 5 | 0.6 | $0.093^{***}$ |
| Number of siblings | | | | | | | | | |
| 0 | 5 | 0.6 | 9 | 1.0 | 3 | 0.3 | 0 | 0.0 | 29.417* |
| 1 | 77 | 8.8 | 134 | 15.4 | 24 | 2.8 | 4 | 0.5 | $0.104^{**}$ |
| 2 | 93 | 10.7 | 181 | 20.8 | 15 | 1.7 | 7 | 0.8 | $0.106^{***}$ |
| 3 | 56 | 6.4 | 106 | 12.2 | 13 | 1.5 | 6 | 0.7 | |
| 4 | 25 | 2.9 | 36 | 4.1 | 4 | 0.5 | 3 | 0.3 | |
| 5 | 19 | 2.2 | 18 | 2.1 | 1 | 0.1 | 0 | 0.0 | |
| 6 | 3 | 0.3 | 10 | 1.1 | 3 | 0.3 | 0 | 0.0 | |
| >7 | 3 | 0.3 | 9 | 1.0 | 4 | 0.5 | 1 | 0.1 | |
| Adaptation in school | | | | | | | | | |
| Yes | 235 | 33.2 | 376 | 53.2 | 49 | 6.9 | 14 | 2.0 | 0.484* |
| No | 10 | 1.4 | 19 | 2.7 | 3 | 0.4 | 1 | 0.1 | $0.922^{**}$ |
| | | | | | | | | | $0.026^{***}$ |
| School grade | | | | | | | | | |
| Poor | 26 | 4.2 | 30 | 4.8 | 6 | 1.0 | 3 | 0.5 | 9.244* |
| Fair | 83 | 13.3 | 123 | 19.7 | 18 | 2.9 | 5 | 0.8 | $0.415^{**}$ |
| Good | 92 | 14.8 | 122 | 19.6 | 20 | 3.2 | 6 | 1.0 | $0.070^{***}$ |
| Excellent | 30 | 4.8 | 56 | 9.0 | 3 | 0.5 | 0 | 0.0 | |
| Mother's BMI | | | | | | | | | |
| Low BMI | 6 | 0.8 | 13 | 1.8 | 3 | 0.4 | 0 | 0.0 | 28.899* |
| Normal | 131 | 17.8 | 187 | 25.4 | 22 | 3.0 | 6 | 0.8 | $\mathbf{0.001}^{**}$ |
| Overweight | 65 | 8.8 | 128 | 17.4 | 27 | 3.7 | 4 | 0.5 | $0.114^{***}$ |
| Obese | 28 | 3.8 | 98 | 13.3 | 10 | 1.4 | 8 | 1.1 | |
| Father's BMI | | | | | | | | | |
| Low BMI | 5 | 0.7 | 14 | 2.0 | 1 | 0.1 | 0 | 0.0 | 45.374* |
| Normal | 134 | 18.7 | 184 | 25.7 | 25 | 3.5 | 3 | 0.4 | $\mathbf{0.000}^{**}$ |
| Overweight | 62 | 8.6 | 161 | 22.5 | 25 | 3.5 | 5 | 0.7 | $0.145^{***}$ |
| Obese | 23 | 3.2 | 54 | 7.5 | 11 | 1.5 | 10 | 1.4 | |

**TABLE 3 (continued)**

| Variables | Low BMI | | Normal | | Overweight | | Obese | | |
|---|---|---|---|---|---|---|---|---|---|
| | n | %† | n | %† | n | %† | n | %† | |
| Meals in a day | | | | | | | | | |
| 1 | 1 | 0.1 | 5 | 0.6 | 0 | 0.0 | 1 | 0.1 | 12.775* |
| 2 | 57 | 6.6 | 116 | 13.4 | 17 | 2.0 | 7 | 0.8 | 0.620** |
| 3 | 190 | 21.9 | 346 | 39.9 | 46 | 5.3 | 11 | 1.3 | 0.070*** |
| 4 | 22 | 2.5 | 33 | 3.8 | 3 | 0.3 | 2 | 0.2 | |
| 5 | 3 | 0.3 | 3 | 0.3 | 0 | 0.0 | 0 | 0.0 | |
| 6 | 3 | 0.3 | 2 | 0.2 | 0 | 0.0 | 0 | 0.0 | |
| Junk meal | | | | | | | | | |
| Yes | 240 | 27.4 | 386 | 44.1 | 47 | 5.4 | 14 | 1.6 | 13.084* |
| No | 42 | 4.8 | 119 | 13.6 | 20 | 2.3 | 7 | 0.8 | **0.004**** |
| | | | | | | | | | 0.122*** |
| TV watching hours | | | | | | | | | |
| 0–2 h/day | 154 | 17.4 | 260 | 29.3 | 44 | 5.0 | 11 | 1.2 | 11.888* |
| 3–4 h/day | 104 | 11.7 | 192 | 21.7 | 17 | 1.9 | 9 | 1.0 | 0.455** |
| 5–7 h/day | 20 | 2.3 | 43 | 4.9 | 4 | 0.5 | 0 | 0.0 | 0.067*** |
| >7 h/day | 6 | 0.7 | 17 | 1.9 | 4 | 0.5 | 1 | 0.1 | |
| Computer use | | | | | | | | | |
| Yes | 238 | 26.9 | 423 | 47.7 | 60 | 6.8 | 18 | 2.0 | 0.972* |
| No | 46 | 5.2 | 89 | 10.0 | 9 | 1.0 | 3 | 0.3 | 0.808** |
| | | | | | | | | | 0.033*** |
| Physical activity‡ | | | | | | | | | |
| Yes | 87 | 10.1 | 193 | 22.4 | 24 | 2.8 | 8 | 0.9 | 4.065* |
| No | 189 | 22.0 | 306 | 35.5 | 42 | 4.9 | 12 | 1.4 | 0.255** |
| | | | | | | | | | 0.069*** |
| Elderly at home | | | | | | | | | |
| Yes | 15 | 1.7 | 36 | 4.2 | 5 | 0.6 | 2 | 0.2 | 1.192* |
| No | 258 | 30.1 | 462 | 53.8 | 61 | 7.1 | 19 | 2.2 | 0.755** |
| | | | | | | | | | 0.037*** |
| Smoking history | | | | | | | | | |
| Nonsmoker | 255 | 28.9 | 434 | 49.2 | 62 | 7.0 | 18 | 2.0 | 7.284* |
| Exsmoker | 25 | 2.8 | 66 | 7.5 | 5 | 0.6 | 3 | 0.3 | 0.295** |
| Regular smoker | 2 | 0.2 | 11 | 1.2 | 1 | 0.1 | 0 | 0.0 | 0.064*** |
| Kovacs CDI | | | | | | | | | |
| Negative | 263 | 29.7 | 455 | 51.4 | 65 | 7.3 | 19 | 2.1 | 4.160* |
| Positive | 21 | 2.4 | 57 | 6.4 | 4 | 0.5 | 2 | 0.2 | 0.245** |
| | | | | | | | | | 0.069*** |
| EAT-40 | | | | | | | | | |
| Negative | 242 | 27.3 | 434 | 49.0 | 60 | 6.8 | 12 | 1.4 | 12.580* |
| Positive | 41 | 4.6 | 78 | 8.8 | 9 | 1.0 | 9 | 1.0 | **0.006**** |
| | | | | | | | | | 0.119*** |

† Total percentage.
‡ PA with the exception of school PA hours.
* Pearson chi-square.
** p value (Bold numbers are found to be statistically significant).
*** Cramer's V.

**TABLE 4**
**Distribution of Study Group According to EAT-40 Results**

| **Variables** | **EAT-40 <30 Points** | | **EAT-40 ≥30 Points** | | |
|---|---|---|---|---|---|
| | **n** | **%†** | **n** | **%†** | |
| Age | | | | | |
| 14 | 40 | 4.5 | 4 | 0.5 | 1.801* |
| 15 | 482 | 54.5 | 94 | 10.6 | 0.406** |
| ≥16 | 226 | 25.5 | 39 | 4.4 | 0.045*** |
| Gender | | | | | |
| Male | 418 | 47.2 | 56 | 6.3 | 10.483* |
| Female | 330 | 37.3 | 81 | 9.2 | **0.001**** |
| | | | | | 0.109*** |
| Mother's education | | | | | |
| Illiterate | 41 | 4.7 | 12 | 1.4 | 5.544* |
| Primary school | 502 | 57.0 | 93 | 10.6 | 0.136** |
| High school | 187 | 21.3 | 26 | 3.0 | 0.079*** |
| University | 14 | 1.6 | 5 | 0.6 | |
| Mother's employment status | | | | | |
| Still working | 103 | 11.7 | 22 | 2.5 | 0.656* |
| Housewife | 609 | 69.0 | 110 | 12.5 | 0.720** |
| Retired | 34 | 3.9 | 5 | 0.6 | 0.027*** |
| Mothly income | | | | | |
| 0-625$ | 299 | 38.3 | 59 | 7.6 | 1.143* |
| 625-1250$ | 296 | 37.9 | 47 | 6.0 | 0.565** |
| >1250$ | 66 | 8.5 | 13 | 1.7 | 0.038*** |
| Number of siblings | | | | | |
| 0 | 13 | 1.5 | 4 | 0.5 | 12.433* |
| 1 | 208 | 23.9 | 31 | 3.6 | 0.087** |
| 2 | 250 | 28.7 | 46 | 5.3 | 0.119*** |
| 3 | 155 | 17.8 | 26 | 3.0 | |
| 4 | 54 | 6.2 | 13 | 1.5 | |
| 5 | 34 | 3.9 | 4 | 0.5 | |
| 6 | 13 | 1.5 | 3 | 0.3 | |
| >7 | 10 | 1.1 | 7 | 0.8 | |
| Adaptation in school | | | | | |
| Yes | 568 | 80.5 | 105 | 14.9 | 3.165* |
| No | 24 | 3.4 | 9 | 1.3 | 0.068** |
| | | | | | 0.067*** |
| School grade | | | | | |
| Poor | 46 | 7.4 | 19 | 3.1 | 11.807* |
| Fair | 195 | 31.4 | 33 | 5.3 | **0.008**** |
| Good | 208 | 33.4 | 32 | 5.1 | 0.138*** |
| Excellent | 79 | 12.7 | 10 | 1.6 | |
| BMI | | | | | |
| Low BMI | 242 | 27.3 | 41 | 4.6 | 12.580* |
| Normal | 434 | 49.0 | 78 | 8.8 | **0.006**** |
| Overweight | 60 | 6.8 | 9 | 1.0 | 0.119*** |
| Obese | 12 | 1.4 | 9 | | |
| Mother's BMI | | | | | |
| Low BMI | 16 | 2.2 | 6 | 0.8 | 10.962* |
| Normal | 307 | 41.8 | 38 | 5.2 | **0.012**** |
| Overweight | 188 | 25.6 | 36 | 4.9 | 0.122*** |
| Obese | 114 | 15.5 | 30 | 4.1 | |
| Father's BMI | | | | | |
| Low BMI | 16 | 2.2 | 4 | 0.6 | 2.677* |
| Normal | 303 | 42.3 | 43 | 6.0 | 0.444** |
| Overweight | 213 | 29.7 | 40 | 5.6 | 0.061*** |
| Obese | 81 | 11.3 | 17 | 2.4 | |

**TABLE 4 (continued)**

| Variables | EAT-40 <30 Points | | EAT-40 ≥30 Points | | |
|---|---|---|---|---|---|
| | n | %[†] | n | %[†] | |
| Meals in a day | | | | | |
| 1 | 4 | 0.5 | 3 | 0.3 | 8.356* |
| 2 | 158 | 18.2 | 38 | 4.4 | 0.138** |
| 3 | 511 | 58.9 | 82 | 9.5 | 0.098*** |
| 4 | 53 | 6.1 | 7 | 0.8 | |
| 5 | 5 | 0.6 | 1 | 0.1 | |
| 6 | 4 | 0.5 | 1 | 0.1 | |
| Junk meal | | | | | |
| Yes | 578 | 66.1 | 108 | 12.4 | 0.081* |
| No | 160 | 18.3 | 28 | 3.2 | 0.438** |
| | | | | | 0.010*** |
| TV watching hours | | | | | |
| 0–2 h/day | 395 | 44.7 | 74 | 8.3 | 6.993* |
| 3–4 h/day | 271 | 30.6 | 50 | 5.6 | 0.072** |
| 5–7 h/day | 62 | 7.0 | 5 | 0.6 | 0.089*** |
| >7 h/day | 20 | 2.3 | 8 | 0.9 | |
| Junk food while TV watching | | | | | |
| Yes | 493 | 58.3 | 98 | 11.6 | 1.748* |
| No | 221 | 26.2 | 33 | 3.9 | 0.111** |
| | | | | | 0.045*** |
| PC use | | | | | |
| Yes | 623 | 70.4 | 115 | 13.0 | 0.036* |
| No | 125 | 14.1 | 22 | 2.5 | 0.482** |
| | | | | | 0.006*** |
| Physical activity[‡] | | | | | |
| Yes | 255 | 29.7 | 57 | 6.6 | 3.214* |
| No | 473 | 55.0 | 75 | 8.7 | **0.046**** |
| | | | | | 0.061*** |
| Physical activity hours | | | | | |
| 0 h/day | 3 | 0.3 | 0 | 0.0 | 16.803* |
| 1–2 h/day | 498 | 56.3 | 74 | 8.4 | **0.002**** |
| 3–5 h/day | 174 | 19.7 | 54 | 6.1 | 0.138*** |
| 6–10 h/day | 57 | 6.4 | 8 | 0.9 | |
| >11 h/day | 16 | 1.8 | 1 | 0.1 | |
| Elderly at home | | | | | |
| Yes | 53 | 6.2 | 5 | 0.6 | 2.258* |
| No | 671 | 78.3 | 128 | 14.9 | 0.088** |
| | | | | | 0.051*** |
| Kovacs CDI | | | | | |
| Negative | 693 | 78.3 | 109 | 12.3 | 23.327* |
| Positive | 55 | 6.2 | 28 | 3.2 | **0.000**** |
| | | | | | 0.162*** |

† Total percentage.
‡ PA with the exception of school PA hours.
* Pearson chi-square.
** p value (Bold numbers are found to be statistically significant).
*** Cramer's V.

**TABLE 5**
**Distribution of Study Group According to Smoking Status**

| | Nonsmoker | | Exsmoker | | Regular Smoker | | |
|---|---|---|---|---|---|---|---|
| | n | % | n | % | n | % | |
| Gender | | | | | | | |
| Male | 375 | 42.5 | 82 | 9.3 | 13 | 1.5 | 49.833* |
| Female | 394 | 44.7 | 17 | 1.9 | 1 | 0.1 | 0.000** |
| | | | | | | | 0.238*** |
| Mother's education | | | | | | | |
| Illiterate | 38 | 4.3 | 12 | 1.4 | 2 | 0.2 | 14.723* |
| Primary school | 524 | 59.7 | 61 | 6.9 | 11 | 1.3 | 0.023** |
| High school | 185 | 21.1 | 25 | 2.8 | 1 | 0.1 | 0.0292*** |
| University | 19 | 2.2 | 0 | 0.0 | 0 | 0.0 | |
| Adaptation in school | | | | | | | |
| Yes | 607 | 86.1 | 58 | 8.2 | 7 | 1.0 | 17.159* |
| No | 23 | 3.3 | 10 | 1.4 | 0 | 0.0 | 0.000** |
| | | | | | | | 0.156*** |
| School grade | | | | | | | |
| Poor | 60 | 9.7 | 5 | 0.8 | 0 | 0.0 | 17.379* |
| Fair | 199 | 32.0 | 26 | 4.2 | 3 | 0.5 | 0.008** |
| Good | 228 | 36.7 | 11 | 1.8 | 0 | 0.0 | 0.118*** |
| Excellent | 87 | 14.0 | 2 | 0.3 | 0 | 0.0 | |
| PC at home | | | | | | | |
| Yes | 293 | 33.2 | 31 | 3.5 | 1 | 0.1 | 7.131* |
| No | 476 | 54.0 | 68 | 7.7 | 13 | 1.5 | 0.028** |
| | | | | | | | 0.090*** |
| Parental smoking | | | | | | | |
| Yes | 322 | 36.7 | 36 | 4.1 | 5 | 0.6 | 1.217* |
| No | 443 | 50.5 | 62 | 7.1 | 9 | 1.0 | 0.544** |
| | | | | | | | 0.037*** |
| Kovacs CDI | | | | | | | |
| Negative | 701 | 79.5 | 86 | 9.8 | 11 | 1.2 | 4.212* |
| Positive | 68 | 7.7 | 13 | 1.5 | 3 | 0.3 | 0.122** |
| | | | | | | | 0.069*** |
| EAT-40 | | | | | | | |
| Negative | 655 | 74.3 | 78 | 8.9 | 12 | 1.4 | 3.348* |
| Positive | 114 | 12.9 | 21 | 2.4 | 1 | 0.1 | 0.188** |
| | | | | | | | 0.062*** |

† Total percentage.
* Pearson chi-square.
** p value (Bold numbers are found to be statistically significant).
*** Cramer's V.

It is also reported that if both parents have high BMI degrees, the obesity risk of the child is 80%; if only one parent has a high BMI degree, the risk decreases to 40%. If neither parent has a high BMI degree, the child still has the risk of obesity with a ratio of 14%[26]. In our study group, the prevalence of obesity with parental obesity was 27.7%, 5.2% with paternal obesity, and 5.5% with maternal obesity.

Eating habits formed in adolescence continue into adulthood; consequently, poor dietary patterns during youth have important implications for health and well-being in adulthood[27]. Adolescents in Turkey spend more eating hours away from home because of additional educational activities, such as attending private teaching institutes and private lessons, so they often skip family meals and prefer junk

**TABLE 6**
**Distribution of Study Group According to Kovacs-CDI Results**

| Variables | KOVACS-CDI <19 Points | | KOVACS-CDI ≧19 Points | | |
|---|---|---|---|---|---|
| | n | %† | n | %† | |
| Age | | | | | |
| 14 | 41 | 4.6 | 3 | 0.3 | 0.786* |
| 15 | 524 | 59.1 | 53 | 6.0 | 0.675** |
| ≥16 | 237 | 26.7 | 28 | 3.2 | 0.030*** |
| Gender | | | | | |
| Male | 446 | 50.3 | 28 | 3.2 | 15.169* |
| Female | 356 | 40.2 | 56 | 6.3 | **0.000**** |
| | | | | | 0.131*** |
| Mother's education | | | | | |
| Illiterate | 46 | 5.2 | 7 | 0.8 | 3.317* |
| Primary school | 537 | 61.0 | 59 | 6.7 | 0.345** |
| High school | 198 | 22.5 | 15 | 1.7 | 0.061*** |
| University | 16 | 1.8 | 3 | 0.3 | |
| Mother's employment status | | | | | |
| Still working | 107 | 12.1 | 18 | 2.0 | 4.875* |
| Housewife | 659 | 74.5 | 61 | 6.9 | 0.087** |
| Retired | 34 | 3.8 | 5 | 0.6 | 0.074*** |
| Mothly income | | | | | |
| 0–625$ | 323 | 41.4 | 35 | 4.5 | 1.253* |
| 625–1250$ | 308 | 39.4 | 36 | 4.6 | 0.535** |
| >1250$ | 74 | 9.5 | 5 | 0.6 | 0.040*** |
| Number of siblings | | | | | |
| 0 | 16 | 1.8 | 1 | 0.1 | 8.876* |
| 1 | 224 | 25.7 | 15 | 1.7 | 0.262** |
| 2 | 267 | 30.6 | 29 | 3.3 | 0.101*** |
| 3 | 162 | 18.6 | 19 | 2.2 | |
| 4 | 60 | 6.9 | 8 | 0.9 | |
| 5 | 31 | 3.6 | 7 | 0.8 | |
| 6 | 13 | 1.5 | 3 | 0.3 | |
| >7 | 15 | 1.7 | 2 | 0.2 | |
| Adaptation in school | | | | | |
| Yes | 612 | 86.6 | 62 | 8.8 | 5.155* |
| No | 26 | 3.7 | 7 | 1.0 | **0.034**** |
| | | | | | 0.085*** |
| School grade | | | | | |
| Poor | 54 | 8.7 | 11 | 1.8 | 4.154* |
| Fair | 208 | 33.4 | 21 | 3.4 | 0.245** |
| Good | 219 | 35.2 | 21 | 3.4 | 0.082*** |
| Excellent | 79 | 12.7 | 10 | 1.6 | |
| Junk meal | | | | | |
| Yes | 613 | 70.1 | 74 | 8.5 | 5.056* |
| No | 178 | 20.3 | 10 | 1.1 | **0.014**** |
| | | | | | 0.076*** |
| TV watching hours | | | | | |
| 0–2 h/day | 426 | 48.1 | 43 | 4.9 | 3.269* |
| 3–4 h/day | 286 | 32.3 | 36 | 4.1 | 0.352** |
| 5–7 h/day | 64 | 7.2 | 3 | 0.3 | 0.061*** |
| >7 h/day | 26 | 2.9 | 2 | 0.2 | |

**TABLE 6 (continued)**

| Variables | KOVACS-CDI <19 Points | | KOVACS-CDI ≥19 Points | | |
|---|---|---|---|---|---|
| | n | %† | n | %† | |
| PC use | | | | | |
| Yes | 672 | 75.8 | 67 | 7.6 | 0.892* |
| No | 130 | 14.7 | 17 | 1.9 | 0.212** |
| | | | | | 0.032*** |
| Physical activity‡ | | | | | |
| Yes | 282 | 32.8 | 30 | 3.5 | 0.005* |
| No | 497 | 57.7 | 52 | 6.0 | 0.517** |
| | | | | | 0.002*** |
| Elderly at home | | | | | |
| Yes | 54 | 6.3 | 4 | 0.5 | 0.509* |
| No | 722 | 84.1 | 78 | 9.1 | 0.331** |
| | | | | | 0.024*** |
| Smoking history | | | | | |
| Nonsmoker | 701 | 79.5 | 68 | 7.7 | 4.212* |
| Exsmoker | 86 | 9.8 | 13 | 1.5 | 0.122** |
| Regular smoker | 11 | 1.2 | 3 | 0.3 | 0.069*** |
| EAT-40 | | | | | |
| Negative | 693 | 78.3 | 55 | 6.2 | 23.327* |
| Positive | 109 | 12.3 | 28 | 3.2 | **0.000**** |
| | | | | | 0.162*** |

† Total percentage.
‡ PA with the exception of school PA hours.
* Pearson chi-square.
** p value (Bold numbers are found to be statistically significant).
*** Cramer's V.

food in order not to spend much time eating. Thus, healthy eating behaviors are often a low priority. In our study group, parents working shift hours was also one important contributing factor. Skipped meals, fewer family meals, and esthetic concerns (especially in girls) at adolescence, result in lower intake of nutrients that lead to low BMI, or higher intake of food groups called "fast food", leading to obesity. According to the present study, it is important to consider "low BMI" as a much more important issue than obesity in developing countries like ours.

In order to gain more time to study, parents usually prefer that their children not take part in PA lessons and school teams. To prepare students for the Nationwide High School Exams and increase their school degree in success lists, even school directorates allow children to study during the PA lesson hours. Also, typically, students on the way to these exams do not want to be on the school teams, and parents or even the directorates themselves do not find them to be beneficial. They consider the hours spent on PA to be a waste of time and think that sports have negative effects on a scholar's success[28]. In a study conducted in the U.S., it was reported that 96.9% of parents supported their children being on school sport teams and 56.3% thought PA increased the success of their students in school[29]. In our study, we found that boys were more active than girls and boys seemed to prefer playing football and basketball games, while girls seemed to prefer volleyball and gymnastics. Our results were similar to a study conducted by Crocker et al.[30]. In addition to the well-known effects, regular PA has many beneficial effects, especially in adolescence. PA increases self-esteem and school success, improves

**TABLE 7**
**Physical Examination Findings According to Gender**

| | Male | | Female | |
|---|---|---|---|---|
| | n | %† | n | %† |
| Strabismus | 1 | 0.1 | 5 | 0.6 |
| Vision impairment | | | | |
| Only in one eye | 22 | 2.5 | 24 | 2.7 |
| In both eyes | 21 | 2.4 | 34 | 3.9 |
| Color blindness | 10 | 1.2 | 0 | 0.0 |
| Hair examination | | | | |
| Dandruff | 40 | 4.6 | 45 | 5.2 |
| Head lice | 1 | 0.1 | 2 | 0.2 |
| Acne | 17 | 2.0 | 19 | 2.2 |
| Tooth decay | 2 | 0.2 | 17 | 2.0 |
| Ear examination | | | | |
| Earwax | 25 | 3.0 | 31 | 3.7 |
| Otitis sequel | 4 | 0.5 | 5 | 0.6 |
| Nose examination | | | | |
| Nasal discharge | 14 | 1.6 | 20 | 2.3 |
| Septal deviation | 9 | 1.0 | 2 | 0.2 |
| Throat examination | | | | |
| Postnasal discharge | 28 | 3.2 | 7 | 0.8 |
| Tonsil hypertrophy | 18 | 2.1 | 23 | 2.6 |
| Neck examination | | | | |
| Palpable thyroid gland | 1 | 0.1 | 3 | 0.3 |
| Lymphadenopathy | 21 | 2.5 | 6 | 0.7 |

† Total percentage.

personal relationships, and decreases the prevalence of depression[31]. Supporting PA, increasing the tone and power of different muscle groups, and acquiring a lifelong habit of PA are very important during adolescence, the period of rapid growth and development. In European countries, 60–90% of adolescents join in PA at least twice a week; male students are more active and PA frequency incrementally increases from 9th grade until 12th grade. It is important to remember that the "history of physical inactivity is the best predictor of future inactivity"[32].

The results of our study suggest that while TV watching is associated with declines in PA among girls, playing PC games is associated with declines in PA among boys. Our results are consistent with existing literature[33,34,35]. Sallis et al suggested that reductions in TV watching hours in association with PA have been shown to prevent or reduce obesity prevalence[34,36]. Reduced TV watching among girls and PC games among boys and increased participation in school sports teams by girls and boys may prevent PA declines in adolescents[36].

Television has a great impact on the development of sedentary lifestyles. Excess TV watching reduces the PA levels of children and leads to social isolation by reducing relationships with "real people", an increase in obesity, and the development of aggressive behaviors[37]. Limiting TV to 1–2 h daily at most is commonly proposed, particularly for programs containing excess violence with guns, smoking, and alcohol. In the U.S., it is reported that 26% of high school students watched at least 4 h of TV and 21% of them played at least 4 h of computer games; this was more frequent in male students[38]. We found, according to the data from the current study, that approximately 75% of the study group had insufficient PA hours and activities involving sedentary behaviors increased gradually.

Studies on psychological symptomatology and obesity have usually characterized the relationship between depression and obesity as unidirectional. Affective disorders can arise from the chronic embarrassment, shame, and guilt engendered by obesity, and obesity may lead to lower self-esteem among adolescents and young adults. It is also important to clarify the onset of depression in relationship to onset of obesity as depression may be the cause and the result of obesity[39]. In the present study, we found the prevalence of depressive symptoms to be 9.5%. Another study, conducted by Oren and Gencdogan, found the prevalence of depressive symptoms in 9$^{th}$ grade students to be 16.94%, and girls were found to have more depressive symptoms than boys, higher than our findings[40]. In a study conducted in Turkey, the prevalence of depression was found to be 10% in the age group older than 15 years[41]. Kulaksizoglu et al. found that girls had more depressive symptoms and eating problems than boys, which was consistent with our study[42]. Erenis et al. found depression, behavioral problems, and decreased self-esteem in clinically obese adolescents[43]. Childhood depression was associated with an increased BMI and depression, and obesity prevalence increased from adolescence to early adulthood[44,45,46]. A prospective study design is required to help distinguish whether a depressed mood is a cause and not just an effect of obesity.

Approximately 20% of high school students smoke cigarettes in Turkey. In 12 million households, parents smoke and 11 million children are exposed to smoke in public places. The most important factors that determine the start of smoking during adolescence is smoking in the household, pressure from friends, and cigarette advertisements. One out of five children between 13 and 15 years of age smokes. Every day, 80,000–100,000 children start smoking, and 50% of people who started smoking during adolescence continued this habit for 15–20 years[9,10,11,12]. A study conducted in Canada showed that a person who smokes in a household is the single significant factor that determines whether an adolescent will start to smoke[46]. According to WHO data, 1.3 billion people smoke cigarettes and 5 million people die due to smoking each year[11].

Children usually try their first cigarette at 6–7 years of age, alcohol at 12 years of age, and drugs generally at 12–14 years of age. Data concerning risky health-related behaviors among adolescents show that the problem of drug and stimulant substance abuse and alcohol consumption in many countries, as well as in Turkey, is common and growing day by day. The smoking ratio among high school students varies from 1.7 to 55%, and it is more prevalent among students in the final grades and male students[10,11,41,47,48]. The prevalence in Bursa was found to be 3.7% in a study performed in adolescents between 11 and 16 years of age in the Bursa region[49].

Smoking is well known, not only for increasing risk factors for some diseases, but also for leading to environmental hazards. Smokers who began smoking in adolescence continue smoking regularly in adulthood, so it is important to take precautions during this period. In the present study, smoking prevalence was found to be lower than other studies conducted in our country. Ogel et al. found that 16.1% of 10$^{th}$ to 12$^{th}$ year students smoked at least once in their lifetime[47]. We found that this 12.8% rate and our low rates may arise from the fact that the questionnaires were completed in classrooms, so they did not tell the truth about smoking.

There are several potential limitations of this study. First, the data were based on self-reports by the adolescents and thus are subject to self-report bias. Adolescents may have misrepresented their actual problem behaviors, such as cigarette smoking. Second, the sample, which was composed of participants from one of the largest towns of Bursa city, should not be viewed as representative of the general population nationwide.

## SUGGESTIONS

Educational institutes play important roles in preserving and improving health. In Turkey, 16 million students in the range of 6–18 years are educated in approximately 60,000 schools. School children are the age group least likely to be referred to health care professionals. Not smoking, healthy diet, and regular physical activities are among the prioritized healthy behavioral targets.

In recent years, in the name of "Educational Reform", there have been many discussions about innovations and problems in the educational system. In this context, one of the most important issues seems to support psychological counseling units in schools.

Schools are very important places in which children spend most of their time until adulthood. Since school children are a huge, but easily accessible, population, schools should be considered as places for teaching basic values in health and scientific knowledge, and to help them to gain the proper skills and behaviors through health education. Sports activities should be encouraged, since they provide considerable benefits to the physical and psychological development of students.

Our results do not reflect the country as a whole; however, it seems that community education programs started in primary schools may prevent problems later on. Health education should be prioritized in schools, since it is quite possible to transfer healthy living behaviors there. Students are open to learning and personality formation. It is also possible to deliver health services to poor children by screening schools regularly.
